# Dynamic balance and explosive strength appears to better explain single leg hop test results among young elite female basketball athletes

**DOI:** 10.1038/s41598-023-31178-7

**Published:** 2023-04-04

**Authors:** Fernando Dominguez-Navarro, Jose Casaña, Borja Perez-Dominguez, Borja Ricart-Luna, Pedro Cotolí-Suárez, Joaquin Calatayud

**Affiliations:** 1 Exercise Intervention for Health Research Group (EXINH-RG), Department of Physiotherapy, University of Valencia, Calle Gascó Oliag 5, 46010 Valencia, Spain; 2 Department of Physiotherapy, Faculty of Health Sciences, European University of Valencia, Valencia, Spain; 3 I+D+I Alqueria LAB Department, Valencia Basket Club, Valencia, Spain

**Keywords:** Health care, Medical research

## Abstract

To analyze the impact of balance, lower-limb explosive strength and anthropometric variables on the result of the hop test in young elite female basketball athletes. Ninety young elite female basketball athletes (13–17 years), without current or recent lower-limb injury, were assessed in the off-season period of July 2021. Single leg hop test, countermovement jump, Y balance test and anthropometric outcomes were assessed. A correlation study and a regression model were performed to investigate the influence of such outcomes and the value of their participation on the hop test results. It was found a low-to-moderate correlation effect size for both countermovement jump (distance and power flight) and Y balance test values (except interlimb outcomes) with the single leg hop test results. Anthropometric outcomes did not show significant correlation (*p* > 0.05). Regression model revealed that for right hop test, countermovement jump values exhibited an adjusted determination coefficient of 0.408, (β = 0.249, *p* = 0.013), For left hop test, again the countermovement jump values (β = 0.229, *p* = 0.025), and left Y balance test values (β = 0.331, *p* = 0.011) jointly obtained an adjusted determination coefficient of 0.263 significant predictive value for countermovement jump outcomes in both right (*β* = 0.249, *p* = 0.013; *β* = 0.301; *p* = 0.031) and left leg (*β* = 0.229, *p* = 0.025; *β* = 0.365, *p* = 0.040), as well as certain Y balance outcomes values. Explosive strength, and dynamic balance although to a lesser extent, appear to be the most relevant physical-functional factors influencing the single leg hop test results among young elite female basketball athletes. These findings may a serve as a basis to implement targeted interventions, such as plyometric and balance training, for an enhancement on functional rehabilitation and reducing the risk of injury related to the hop test results.

## Introduction

The practice of sports carries an intrinsic risk of suffering sport injuries, knowing that this risk, due to neuromuscular, biomechanical and hormonal reasons, is greater in female athletes^1,2^. In female basketball, lower-limb injuries are the most prevalent, with an increase in the number of cases reported during the last decade^3^. This situation entails negative aspects for the athlete on a physical and psychological level^4^, whereas the issue becomes even more important in young athletes, as the injury could interfere with their physical and professional development^5^.

Detecting athletes at higher risk of injury is the first step in the injury prevention process. The assessment of the physical-functional status becomes crucial for this objective, serving both as a starting point for designing a conditioning injury prevention program and to monitor recovery^6^. Indeed, the hop test is a widely used tool to assess the functional capacity of the lower limb, requesting a propulsive action to perform a horizontal jump and landing^7^. Its results have a functional implication, since they are useful in predicting the risk of lower limb injuries^8^, detecting functional improvements^9^, and guiding return-to sport decisions^10^. The study of its psychometric properties has demonstrated that it is a valid and reliable tool for functional assessment^11,12^, which has led to its recommended used in the field of sports in the context of both prevention and recovery from injuries^7^.

As a functional test, the hop test has a global approach where different systems and physical capacities will be required and integrated for its performance. Deficiencies in the neuromuscular and biomechanical systems may lead in abnormal hop patterns, which requires a segmented study of these factors to better elucidate interpretation of hop test results.

Thigh muscle strength is one of the most studied influential factors. Specifically, peak knee flexors and extensors torque and rate of torque development are shown to positively correlate to hop distance outcomes^13–15^. Discussed issues of this assessment include that the force produced is in open kinetic chain, unlike most sporting activities; and that the joint angulation in the measurement influences the results^13^. Instead of an analytic evaluation, the measurement of lower limb explosive strength, evaluated with maneuvers such as the vertical jump, has been proposed as an alternative for its analysis, with studies revealing a positive correlation between the vertical and longitudinal jump distances achieved^16^. However, muscular strength alone does not seem to account for the dynamic stability necessary to hop effectively^17^, so dynamic balance is also required. Dynamic balance can be understood as the ability to keep the center of gravity stable in situations of movement, such as hop distance. Previous research indicates that diminished ability for dynamic balance induces altered hop patterns^18^. In addition, the biomechanics of this movement are also influenced by the anthropometric characteristics of the athlete. Different levels of BMI and leg length appear to vary the lever arms and moments exerted on the lower extremities during jumping^19^. Furthermore, it is not entirely clear how these factors specifically influence female basketball athletes, as these characteristics have been found to differ from those of athletes of the same age who practice other sports^20^.

Considering the hop test as a global movement, which involves the state and functioning of different neuromuscular and biomechanical aspects, little is known about the degree of participation of each of these elements and their ultimate implication on the test results. Therefore, it is relevant to study the impact of these physical capacities on its execution for a better understanding of its functional composition. The aim of this research is to analyze the impact of dynamic balance, lower-limb explosive strength and anthropometric outcomes on the result of the hop test in young elite female basketball athletes. We hypothesized that these physical-functional capacities, as well as leg length, will contribute to the results on the hop test.

## Methods

### Study design

This is a cross-sectional study carried out in June 2021, during the off-season period, at the Alquería del Basket of Valencia Basket facilities, where included aprticipants underwent a functional evaluation for anthropometric, neuromuscular and biomechanics outcomes and it was analyzed their relation to the single leg hop test.

### Subjects

To have a homogeneous and representative sample of trainee-level elite female basketball athletes, subjects between 13 and 17 years old, belonging to the youth academy of different Spanish women's basketball first division were invited to participate in the study. A total of 90 athletes meeting these criteria agreed to participate in the study. Exclusion criteria to participate in the study was: (I) current acute injury receiving treatment, (II) non-surgical injuries to the lower limbs within the last 3 months; (III) surgical injuries to the lower limbs within the last 9 months; (IV) any other physical or psychological condition that altered the physical and functional status of the athletes, those conditions being based on the athlete's self-report or detected by the research staff.

Participants and parents/legal guardians gave their written informed consent to participate in the study, in accordance with the ethical guidelines of the Declaration of Helsinki and subsequent updates. This study obtained the ethical approval of the Ethics Committee of the Universitat de Valencia (UV-INV_ETICA-1523453). The research was developed and reported according to the recommendations of STrengthening the Reporting of OBservational studies in Epidemiology (STROBE)^21^.

### Procedure

The outcome assessment was performed in the aforementioned facilities by two physical therapists, with experience in the evaluation of these tests. Evaluations were all performed at the same day and by using the same equipment. Age and anthropometric data were obtained first for all participants. Before physical testing, athletes performed a 10-min general warm-up, consisting of medium-intensity aerobic exercises, mobility, and coordination. The physical tests were performed in the same order: Counter movement jump (CMJ), Y balance test (YBT) and single leg hop test, with a 2-min rest between them. Athletes were wearing sports clothes and shoes on. All tests were verbally explained, and a couple of attempts for each leg were allowed without recording results, to be familiarized with the test. Three valid measurements were taken from each test evaluation and the best one was registered.

### Outcomes

#### Counter movement jump

**Figure 1:**
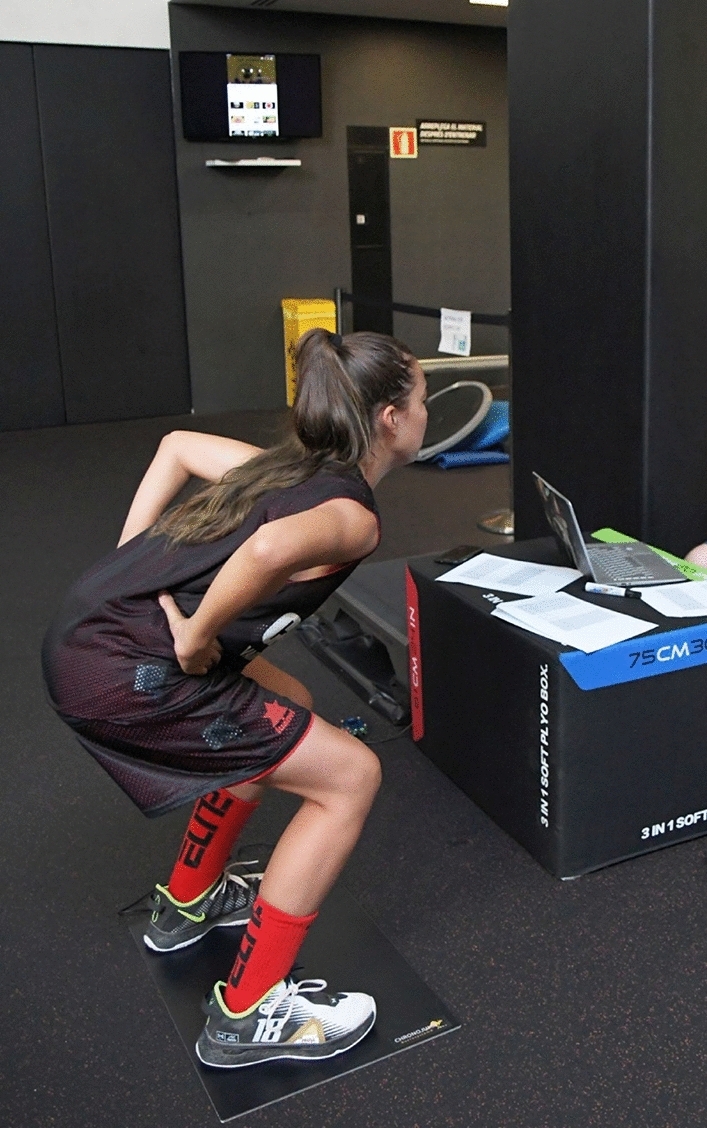
Procedure of the Countermovement jump test.

To measure explosive strength, the CMJ test was used. To perform the test, participants initially stood on the platform, adopting a position of equal weight distribution between legs. Then, they were asked to perform a fast vertical jump, as high as possible, with both arms resting on the hips to avoid arm swing (Fig. 1). The data was collected with the Chronojump contact platform equipment, (Boscosystem®, Barcelona, Spain), registering the flight height (cm) and power (watts). The CMJ has proven extremely high reliability (ICC = 0.95)^22^, as well as Chronojump contact platform is considered a suitable equipment for its evaluation in elite athletes^23,24^.

#### Y balance test

This is a validated simplified version of the original Star Excursion Balance Test, in which 3 of the 8 components of the originally proposed reaching directions (anterior displacement, postero-medial displacement and posterolateral displacement) are assessed. The test has reported excellent inter and intra-rater reliability scores (ICC of 0.88 and 0.90, respectively) in healthy population^25^, and it is commonly used for the assessment of female basketball players^26^. YBT is described as a dynamic balance assessment tool, although it also requires mobility, motor control and coordination of the lower limb.

**Figure 2:**
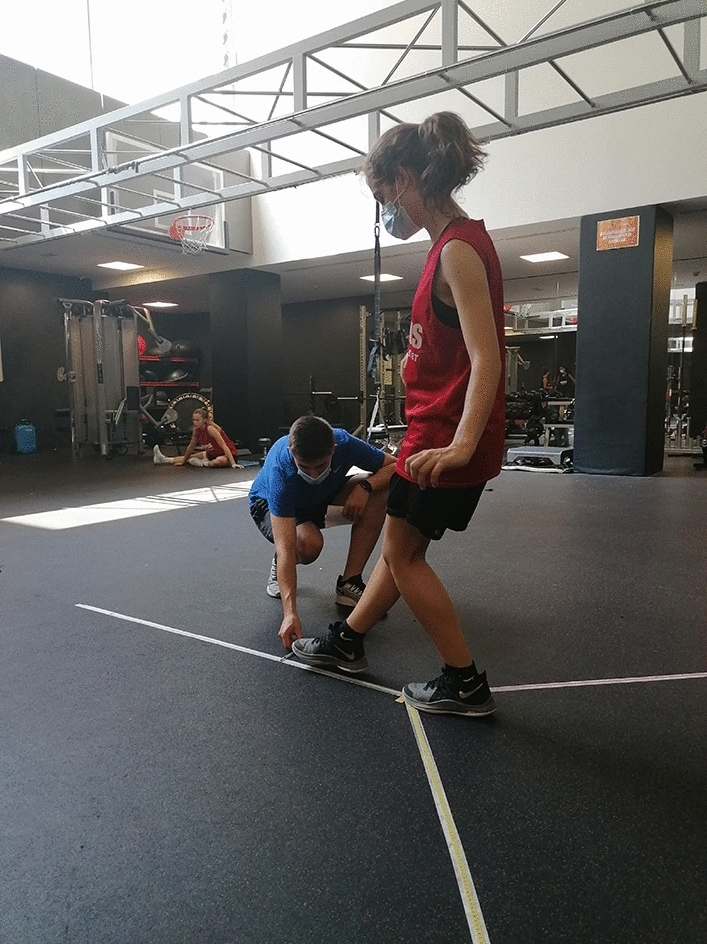
Y balance test procedure.

To perform the test, three lines of tape were laid out on the floor so that one formed the anterior axis, and the other two were arranged at 135 degrees to it. Participants were asked to adopt a double-legged starting position, at the intersection of the 3 lines. The participants were asked to extend the contralateral leg to the one being assessed along one of the 3 components of the directions, as far as possible, without losing balance or altering the support position of the assessed leg (Fig. 2). Once the measurement of the 3 directions was completed, the same protocol was repeated with the opposite leg. The mean score for each leg was obtained by adding the scores of the three components and dividing by 3. The difference between the scores obtained for each leg was also calculated and expressed as a percentage (YBT interlimb difference).

#### Single-leg hop test

This functional test has proved to have excellent reliability, with intraclass correlation coefficients ranging from 0.92 to 0.96^27,28^. The test was assessed according to the methodology described by Noyes et al.^29^. A tape measure was placed on the ground, parallel to the surface set up for the jump. Athletes were asked to stand, first, on the dominant limb and perform a maximum-effort forward jump, landing on the same limb, without losing balance, changing the support surface, or marking extra supports for at least 3 s. If these criteria were not met, the test was considered invalid. When the test was valid, the distance covered by the jump was registered, expressed in centimeters. Hop symmetry index was calculated through: hop distance on the dominant limb / hop distance non-dominant limb.

### Data analysis

Descriptive data included mean and standard error of mean of the used outcome. The normal distribution of data was checked the Kolmogorov–Smirnov test. A correlation study was performed between the single-leg hop test assessment (independent variable), and the rest of physical tests, anthropometric and age of participants, acting as dependent variables. Pearson’s R was used to explore the correlation of each of the dependent and independent parameters. The correlation effect size was interpreted as follows:  < 0.1, trivial; 0.11–0.3, low; 0.31–0.5, moderate; 0.51–0.7, large; 071, − 0.9, very large; > 0.9, almost perfect^30^. A linear regression model for dependent variables was created with all variables that exhibited significant correlations. Statistical data analysis was conducted using SPSS v26 (Inc., Chicago, IL, USA).

### Informed consent for image publication

Informed consent from all subjects and/or their legal guardian(s) was obtained for publication of identifying information/images in an online open-access publication.

## Results

Ninety healthy female basketball athletes (mean age: 15.1 (± 1.2) years) participated in the study. Table 1 show the descriptive values of the assessed results in the total sample, with the results of the anthropometric outcomes revealing athletes measure a mean of 1.69 (± 0.1) meters, weigh 58.33 (± 8.4) kg, and have a leg length of 0.84 (0.6) meters.

**Table 1:** Descriptive values for the total sample.

Outcomes	Total sample (n = 90)
	Mean (SEM)
Anthropometric variables	
Age (years)	15.1 (1.22)
Height (m)	1.69 (0.11)
Weight (kg)	58.33 (8.40)
Body Mass Index	20.35 (2.63)
Leg length	0.84 (0.60)
Dominant leg (n (percentage))	
Right	82 (91.1%)
Left	8 (8.9%)
Single leg hop test	
Right leg (cm)	170.12 (25.6)
Left leg (cm)	169.86 (27.5)
Limb Symmetry Index (%)	− 0.41 (20.69)
Physical function variables	
Right YBT mean score (cm)	70.64 (9.32)
Left YBT mean score (cm)	70.41 (6.14)
Inter-limb YBT difference (%)	0.13 (0.10)
CMJ flight distance (cm)	22.45 (2.50)
CMJ flight power (w)	637.26 (78.08)

YBT: Y Balance Test; CMJ: Counter Movement Jump.

The results derived from the correlation study and predicting model scores represents a higher contribution of the lower-limb explosive strength (CMJ values) to the hop test results, as well as a lower contribution of the dynamic balance (YBT-derived values). No significant relevance was found for the anthropometric outcomes in hop test results.

Table 2 shows the results for the correlation study. Significant correlation values were found for the YBT mean scores and the single leg hop test (both ipsilateral and contralaterally), with effect sizes that ranged from low to moderate (Right YBT mean score with right and left hop test: Pearson = 0.211 and 0.407; *p* = 0.046 and < 0.001, respectively) (Left YBT mean score with right and left hop test: Pearson = 0.410 and 0.244; *p* =  < 0.001 and 0.043, respectively). Likewise, from CMJ values, flight distance exhibited moderate effect size correlations with right (Pearson = 0.331; *p* = 0.001) and left hop test (Pearson = 0.313; 0.003) as well as flight power (Pearson = 0.385; *p* = 0.001; Pearson 0.367; *p* = 0.001). Correlations with anthropometric outcomes were not significant (*p* > 0.05).

**Table 2:** Correlation study for the total sample of the study (n = 90). Pearson correlation (*p* values).

	Right single leg hop test	Left single leg hop test	Hop test interlimb
Dynamic balance outcomes			
Right YBT mean score	0.211 (0.046)*	0.407 (< 0.001)*	− 0.030 (0.783)
Left YBT mean score	0.410 (< 0.001)*	0.244 (0.043)*	0.061 (0.569)
YBT interlimb	− 0.129 (0.232)	− 0.130 (0.224)	− 0.129 (0.232)
Lower-limb Explosive force outcomes			
Flight distance (CMJ)	0.331 (0.001)*	0.313 (0.003)*	0.097 (0.367)
Flight Power (CMJ)	0.385 (0.001)*	0.367 (0.001)*	0.061 (0.438)
Anthropometric outcomes			
Length leg	0.183 (0.083)	0.103 (0.332)	0.070 (0.846)
Age	0.172 (0.104)	0.072 (0.502)	0.043 (0.687)
Height	0.124 (0.256)	0.107 (0.303)	0.081 (0.547)
Weight	0.034 (0.747)	0.018 (0.865)	0.035 (0.744)
BMI	− 0.112 (0.292)	− 0.058 (0.590)	0.067 (0.532)

Significant values are in bold.

YBT: Y Balance test; CMJ: Counter Movement Jump, BMI: Body Mass Index. * indicates *p* vales < 0.05.

The predicting model scores, displayed in Table 3, show that for right hop test, CMJ flight distance and power exhibited an adjusted determination coefficient of 0.408, (β = 0.249, *p* = 0.013), being of the outcomes analyzed, the ones that best explain the results for right hop test. For left hop test, again the CMJ values (β = 0.229, *p* = 0.025), and left YBT (β = 0.331, *p* = 0.011) jointly obtained an adjusted determination coefficient of 0.263.

**Table 3:** Predicting model values for dependent variables.

Dependent variable	R^2^adj	Predictor	β	*p* value
Hop test right	0.408	YBT right	− 1.465	0.565
		YBT left	2.025	0.472
		CMJ	0.249	0.013*
Hop test left	0.263	YBT right	− 0.023	0.850
		YBT left	0.331	0.011*
		CMJ	0.229	0.025*

Significant values are in bold.

YBT: Y Balance test; CMJ: Counter Movement Jump. * indicates *p* vales < 0.05.

## Discussion

The hop test is a widely used tool for the functional assessment in athletes due to its easy and low-cost implementation, as well as its functional relevance^9,28^. The main findings of the present study revealed that mainly CMJ-derived parameters, flight distance and power, determine the results of the hop test; as well as, to a lesser extent, dynamic balance levels. This contributes to a better understanding of the physical-functional components of the hop test and the interpretation of its results, highlighting that it has a multifactorial and not a unidimensional component, since different physical-functional aspects are integrated for its execution. Coaches, trainers and medical staff can benefit from these results, as it helps to design specific strategies for the improvement of these capacities, which will have an impact on the prevention of injuries and functional improvement of athletes.

Regarding dynamic balance, the results of the study demonstrated a correlation for the YBT with the hop test scores, both between the ipsilateral and the contralateral leg. Balance and postural control are key elements in the performance of functional movements in sport, and the alteration of these abilities has a negative impact on functional performance. In turn, dynamic balance, has been pointed out as a relevant factor in the prevention of sports injuries^31^. The capacity of dynamic balance would therefore be involved in the performance of the hop test, since a horizontal displacement of the center of masses will be produced, being necessary a correct stabilization process, especially during the flight and landing phase. In addition, stress on the dynamic balance will be greater as it employs a one-legged landing. Furthermore, it has been suggested that the neuromuscular activity in vastus medialis and biceps femoris required to perform the hop test is similar to the one that which occurs during dynamic stabilization activities^32,33^. Boey & Lee highlighted that those higher levels of dynamic stability improve the biomechanics of landing in a one-legged hop^34^. Likewise, Paterno et al. analyzed single-leg dynamic stability and postural control through a dynamometric platform, also suggesting the importance of those capacities for a greater achieved distance in the hop test^35^, which would be in line with our results. However, the results obtained in the regression model limit the impact of dynamic balance as a component of the single leg hop test.

On the other hand, CMJ evaluates the explosive strength of the lower limb by requesting a stretch shortening cycle action to generate propulsive strength in a vertical jump. The vertical distance reached during the jump and the power generated are indicators of such explosive strength. We found a moderate correlation between the outcomes obtained in the CMJ and the single leg hop test. Moreover, the regression model verified that both CMJ flight distance and power influence the hop test results, and therefore, it could be postulated that explosive strength acts as a relevant physical-functional contributor to the results obtained in the single leg hop test. The relationship of lower-limb strength and the hop test has been analyzed primarily from isokinetic devices to assess muscle strength, with most of these studies revealing positive correlations^13,15,36^. The CMJ offers another way to evaluate components of strength, with the main advantage that the generated strength is produced in a closed kinetic chain, and recreating a more functional movement, as well as with greater transference to sports activities in basketball. Therefore, the results of this study would complement those that, by means of isokinetic analysis, have reported a positive correlation between lower limb muscle strength and the results obtained in the hop test, offering a different dimension to evaluate strength, based on the stretch shortening cycle action.

Considering the physiology of the movement, we observe that both in the hop test and CMJ the movement is produced from the plyometric contraction of the lower limb, although with perpendicularly different vectors. This similarity in gesture could explain the influence of explosive strength, measured in a vertical jump, on the results of the hop test. But despite this apparent similarity, other authors point out that the physical demands experienced in both tests are different^37^, reporting also different values in some cases, suggesting that the learning processes of the two types of jumps are different during the maturational stage^38^. Specifically, some authors point out that hop test requires a more complex neuromuscular strategy to ensure stability during forward displacement^37^ as well as others advocate that the physical demand is greater in the CMJ^39^, so it has also been granted to be more sensitive when detecting functional deficiencies^40^.

Based on the contribution of balance and explosive strength on the results of the hop test, it can be hypothesized that specific strategies to improve these conditions, such as balance or plyometric training will enhance the results of this test, and therefore influence the risk of injury and its functional capacity, as suggested in previous studies^26,41^.

Age, perhaps surprisingly, was not found to significantly determine strength or balance levels in the subjects assessed. Participants in the present study age between 13 and 17 years old, so they are still in the maturation process. The maturation of the musculoskeletal system at these ages leads to an increase in physical capacities, such as muscle strength^42^. However, other studies challenge this assumption, such as the one conducted by Barber-Westin on 853 female athletes between 9 and 17 years of age, which found no consistent evidence of a linear relationship between the two factors^43^. Therefore, although the chronological age of athletes seems to influence muscle strength levels, there are other maturational, biomechanical, and training aspects that are also relevant when explaining the evolution of physical capacities such as strength or dynamic stability^44^. Precisely, the current literature debates the suitability of taking into account maturational age (through formulas such as peak height velocity) to the detriment of chronological age in studies carried out in pediatric and adolescent population^45^.

The present analyses found that anthropometric outcomes were not statistically significant in the hop test result. Although it has been proposed that the results of the hop test should be expressed in relation to the length of the lower limb^46^, the findings of the present study did not certify that assumption. A longer leg length means that a greater horizontal distance may be achieved. However, a greater lever arm in the lower limbs may also mean changes in neuromuscular activation patterns and biomechanics during jumping^19^. Even more so in maturational processes, where rapid growth can lead to greater adaptations having to be made. Yet this aspect should be further explored in future studies, it may be that at maturing ages, a greater length of the lower limbs does not necessarily entail a greater capacity for horizontal jumping.

Interlimb-hop differences are other common expression of the hop test results, due to its capacity to detect functional differences^9^. However, in the present study, no variables related to this result were found.

### Strengths and limitations

Among the strengths of the study, it should be noted that the assessment was performed among a specific sample of young elite basketball players, with a considerable sample size. Furthermore, sophisticated assessment equipment, such as the Chronojump contact platform, was used.

However, the study presents certain limitations that should be considered. First, the influence of certain outcomes has been analyzed, focusing on those related to balance, explosive strength, and anthropometric factors, ignoring others that may also be relevant. Calculating maturational age of participants according to peak height velocity formula may be an interesting aspect for further studies. In addition, the cross-sectional nature of the study does not allow to stablish cause-effect or extrapolation the results to different moments of the season.

## Conclusions

Explosive strength, and dynamic balance although to a lesser extent, appear to be the most relevant physical-functional factors influencing the single leg hop test results among young elite female basketball athletes. These findings may a serve as a basis to implement targeted interventions, such as plyometric and balance training, for an enhancement on functional rehabilitation and reducing the risk of injury related to the hop test results.

## Data Availability

Data available on request to corresponding author due to privacy/ethical restrictions.
